# Developing a network view of type 2 diabetes risk pathways through integration of genetic, genomic and functional data

**DOI:** 10.1186/s13073-019-0628-8

**Published:** 2019-03-26

**Authors:** Juan Fernández-Tajes, Kyle J. Gaulton, Martijn van de Bunt, Jason Torres, Matthias Thurner, Anubha Mahajan, Anna L. Gloyn, Kasper Lage, Mark I. McCarthy

**Affiliations:** 1 Wellcome Centre for Human Genetics, University of Oxford, Oxford, UK; 2 Department of Pediatrics, University of California, San Diego, CA USA; 3 Oxford Centre for Diabetes, Endocrinology and Metabolism, University of Oxford, Oxford, UK; 4 Oxford NIHR Biomedical Research Centre, Churchill Hospital, Oxford, UK; 5 Department of Surgery, Massachusetts, General Hospital, Boston, MA USA; 6 Broad Institute of MIT and Harvard, Cambridge, MA USA; 7 Harvard Medical School, Boston, MA USA; 8 Present Address: Department of Bioinformatics and Data Mining, Novo Nordisk A/S, Maaloev, Denmark

## Abstract

**Background:**

Genome-wide association studies (GWAS) have identified several hundred susceptibility loci for type 2 diabetes (T2D). One critical, but unresolved, issue concerns the extent to which the mechanisms through which these diverse signals influencing T2D predisposition converge on a limited set of biological processes. However, the causal variants identified by GWAS mostly fall into a non-coding sequence, complicating the task of defining the effector transcripts through which they operate.

**Methods:**

Here, we describe implementation of an analytical pipeline to address this question. First, we integrate multiple sources of genetic, genomic and biological data to assign positional candidacy scores to the genes that map to T2D GWAS signals. Second, we introduce genes with high scores as seeds within a network optimization algorithm (the asymmetric prize-collecting Steiner tree approach) which uses external, experimentally confirmed protein-protein interaction (PPI) data to generate high-confidence sub-networks. Third, we use GWAS data to test the T2D association enrichment of the “non-seed” proteins introduced into the network, as a measure of the overall functional connectivity of the network.

**Results:**

We find (a) non-seed proteins in the T2D protein-interaction network so generated (comprising 705 nodes) are enriched for association to T2D (*p* = 0.0014) but not control traits, (b) stronger T2D-enrichment for islets than other tissues when we use RNA expression data to generate tissue-specific PPI networks and (c) enhanced enrichment (*p* = 3.9 × 10^− 5^) when we combine the analysis of the islet-specific PPI network with a focus on the subset of T2D GWAS loci which act through defective insulin secretion.

**Conclusions:**

These analyses reveal a pattern of non-random functional connectivity between candidate causal genes at T2D GWAS loci and highlight the products of genes including *YWHAG*, *SMAD4* or *CDK2* as potential contributors to T2D-relevant islet dysfunction. The approach we describe can be applied to other complex genetic and genomic datasets, facilitating integration of diverse data types into disease-associated networks.

**Electronic supplementary material:**

The online version of this article (10.1186/s13073-019-0628-8) contains supplementary material, which is available to authorized users.

## Background

The rising prevalence of type 2 diabetes (T2D) represents a major challenge to global health [1]. Current strategies for both prevention and treatment of T2D are suboptimal, and greater insight into the mechanisms responsible for the development of this condition is a prerequisite for further advances in disease management [2].

The identification of human DNA sequence variants which influence predisposition to T2D provides one of the most direct approaches for deriving mechanistic insight. However, current understanding of the genetic architecture of T2D indicates that the genetic component of T2D predisposition likely involves variation across many thousands of loci [3, 4]. Close to 500 independent genetic signals for which there is robust evidence of a contribution to T2D predisposition have been identified, largely through genome-wide association studies, supplemented by analysis of exome- and genome-sequence data [4–6]. This profusion of genetic signals has raised questions concerning the extent to which the inherited susceptibility to complex traits such as T2D can be considered to occupy finite biological space [7]. In other words, as the number of loci influencing T2D risk increases, will the mechanisms through which these are found to mediate the development of this condition continue to proliferate, or will they start to converge around a limited set of pathways?

There are two main challenges in addressing this key question. First, whilst a minority of the causal variants underlying these association signals are coding (and therefore provide direct inference regarding the genes and proteins through which they act), most lie in regulatory sequence. This makes assignment of their effector transcripts a non-trivial exercise and obscures the downstream mechanisms through which these variants impact T2D risk [8–10]. This challenge can increasingly be addressed through the integration of diverse sources of relevant data including (a) experimental data (e.g. from studies of cis-expression or conformational capture) which link regulatory risk variants to their likely effectors [11, 12] and (b) evaluation of the biological evidence connecting each of the genes within a GWAS-associated region to the disease of interest. In the present study, focussing on a set of approximately 100 T2D risk loci with the largest effects on T2D predisposition, we use a range of information to derive “positional candidacy” scores for each of the coding genes mapping to T2D-associated GWAS intervals.

The second challenge lies in the requirement to define functional relationships between sets of candidate effector transcripts in ways that are robust and, in particular, orthogonal to the data used to assign candidacy in the first place [13, 14]. Solutions for the second challenge are less well-developed but generally involve some type of network analysis (e.g. weighted gene correlation network analysis [WGCNA]) and application of the “guilt-by-association” framework to infer function [15–17]. However, recourse to co-expression information or functional pathway enrichment methods to generate and evaluate such networks runs the risk of introducing circularity, given that information on expression and function typically contributes (whether explicitly or not) to assignments of effector transcript candidacy. The use of protein-protein interaction data provides one possible solution to this conundrum [18]. In the present study, we make use of external protein-protein interaction data from the InWeb3 dataset [19, 20] to evaluate and characterise the connectivity of the T2D candidate effector transcripts in terms of their ability to nucleate empirically confirmed interactions between their encoded proteins.

## Methods

### Positional candidacy score derivation

We developed a framework to score the candidacy of genes mapping to GWAS association signals which aggregated data from multiple sources. The information collected fell into two categories. First, we used regression-based approaches to link disease-associated variants (most of which map into non-coding sequence and are therefore presumed to act through transcriptional regulation of nearby genes) to their likely effector transcripts, using a combination of variant-based annotations and expression QTL data [21]. We combined the two measures to generate a “positional candidacy score” (PCS) for each gene. We applied this framework to 1895 genes located within a 1Mb interval around the lead variants from 101 T2D GWAS regions. These represent the loci with the largest effect sizes for T2D, as identified in European subjects as of early 2017 [4, 6, 22] (Additional file 1: Table S1).

#### Mapping effector transcripts to GWAS signals

At each of the 101 loci, we collected summary T2D case-control association data (−log_10_ *p* values) for all 1000 Genomes variants in the 1-Mb interval surrounding the lead variants [6]. We then annotated the most associated variants in each interval using gene-based annotations for all genes in the interval from several sources. First, we collected relevant discrete annotations for all protein coding genes in GENCODE (version 19) [23] within the interval including (a) coding exon location, (b) promoter location (defined as 1-kb region upstream of the transcription start site [TSS]) and (c) distal regulatory elements correlated with gene activity from DNAseI hypersensitivity (DHS) data (ENCODE version 3) [24]. We assigned each variant a binary value based on whether it overlapped one of the discrete annotations for a gene in the interval (exon, promoter, distal element). Second, we collected summary statistic expression QTL (eQTL) data from liver, skeletal muscle, whole blood, subcutaneous adipose and visceral adipose (GTEx version 6) [21] and pancreatic islets [11]. We assigned each variant the −log_10_ *p* value of eQTL association for each cell type for each gene in the interval. Third, we calculated the distance of each variant to the TSS of each gene in the interval and assigned each variant the inverse TSS distance for each gene (i.e. variants closer to the TSS have higher values). Variants without values in the eQTL datasets were removed from the analysis.

We then performed feature selection for each T2D locus separately using elastic net regression (R package glmnet) with the T2D *p* values as the outcome variable and binary genomic annotations (exon, promoter, distal element), distance to TSS and cell type cis-eQTL *p* values for each gene in the interval as the predictor variables. We also included minor allele frequency and imputation quality of each variant at the locus as predictor variables. We obtained the effects of features selected from the resulting model. We applied a 10-fold scaling factor to coding exon features, based on known enrichment of T2D variants in coding exons [25, 26]. Where multiple features were selected for the same gene (e.g. distal DHS site and tissue eQTL), we summed the effects for that gene. We considered the summed effects of features for each gene as the “variant link score” in subsequent analyses.

#### Semantic mapping of gene functional annotations

We also derived a second score of the T2D relevance for each gene within the 101 GWAS intervals based on the annotations for each within data from Gene Ontology (GOA, version 157), the Mouse Genome Database (MGD, version 6.08), and biological pathways (KEGG) (version 83.1), compiling these annotations into a single document per gene. We also created a query document of empirically compiled terms we considered relevant to T2D pathophysiology (listed here: https://github.com/kjgaulton/gene-pred/blob/master/res/T2D.query.manual.txt). Both gene documents were converted into a word matrix. We calculated the total number of unique words across all documents *N*, after removing a list of commonly used “stop” words from PubMed (https://www.ncbi.nlm.nih.gov/pubmed/) and stemming the remaining words. We weighted each word *w* for each gene document *g* using “term frequency (TF)” minus “inverse document frequency” defined as:

$$ \mathrm{TF}={f}_{g,w} $$

$$ \mathrm{IDF}=\log \left(\frac{N}{n_w}\right) $$

where *n*_*w*_ is the number of documents containing word *w.* We defined the value (*g*_w_) of word *w* in gene document *g* as:

$$ {g}_w=\mathrm{TF}\times \mathrm{IDF} $$

and applied latent semantic analysis (LSA) using singular value decomposition of the weighted matrix *M*

$$ M= TS{D}^T $$

where *T* is the left singular vector matrix of terms, *D* is the right singular vector matrix of documents, *S* is the diagonal matrix of singular values, and the number of dimensions was determined by the function *dimcalc_share* from the *lsa* package [27]. We used the resulting matrices to identify genes with functional attributes that indicated relevance to the T2D pathogenesis. For each gene document vector *g*, we calculated similarity scores *S*_*i,q*_ using the dot product between the gene vector and the T2D query vector *q*

$$ {S}_{g,q}=g\bullet q $$

From these data, we extracted similarity scores for the 1895 genes of interest, which we considered the “semantic score” in subsequent analyses.

#### Combining gene scores

For each of the 1895 genes, we scaled scores from these two analyses to the sum of scores for each of the *x* genes at each locus resulting in a semantic score *s*_*g*_ and variant link score *v*_*g*._ To calculate a positional candidacy score (PCS), we averaged the two scores and rescaled across all *x* genes at each locus.

$$ {CS}_g=\frac{s_g+{v}_g}{\sum_{i=1}^x{s}_i+{v}_i} $$

### Network modelling

#### Selection of the “seed node set”

At each GWAS locus, we defined the sets of genes that, after ranking the genes for each locus by decreasing PCS, generated a cumulative PCS exceeding 70%. This reduced the set of 1895 genes of interest to 451 “seed” nodes for subsequent network analysis. We performed network analyses using an updated version of InWeb3, a previously described comprehensive map of protein-protein interactions, containing 169,736 high-confidence interactions between 12,687 gene products compiled from a variety of sources [19, 20]. We updated the version used in [20], by updating outdated gene symbols and restricting interactions to those deemed “high confidence” (score > 0.124).

#### Prize-collecting Steiner tree formulation

We formulated the task of examining the connectivity of GWAS positional candidates (the set of 451 “seed” genes) within protein-protein interaction space as an asymmetric prize-collecting Steiner tree (APCST) problem. APCST-like approaches have been widely used to solve network-design problems [28–30]. The APCST seeks to connect “seed” nodes (in formal nomenclature, “terminals”) to collect “prizes”, using confirmed protein-protein interactions as edges. Prizes are weights added to seed nodes: in our analysis, these correspond to the PCS values for each “seed” gene, derived from the -omic integration approach. “Linking” (formally, “Steiner”) nodes (that is, proteins/genes not included in the seed set) can be introduced into the network, where necessary. Network expansion is controlled by the balance between the benefits of adding a particular node (increased connectivity between seed genes, driven by the collection of prizes) vs. the costs of adding additional edges (based on a function which penalises expansion of the network). In mathematical terms, we defined the APCST as follows: given a directed graph *G* = (*V*, *A*), arc costs *c*: *A* ⟼ *ℝ* ≥ 0, node prizes *p*: *V* ⟼ *ℝ* ≥ 0 and a set of fixed terminals *T*_*f*_, the goal is to find an arborescence *S* = (*V*_*s*_, *A*_*s*_) ⊆ *G* that spans *T*_*f*_ such that the following function is maximised:

$$ P(S)=\beta \sum \limits_{i\in {V}_s}{p}_i-\sum \limits_{\left(i,j\right)\in {A}_s}{c}_{i,j} $$

In this formulation, we reward the inclusion of nodes *i* ∈ *V*_*s*_ with higher prizes (that is, higher PCS values) (first term of equation) whilst paying costs for including edges (second term of equation). The parameter, *β*, scales the importance of node prizes versus edge costs in the optimization and can be used to titrate the size of the generated network. We tested different values of *β* (between 4 and 30) and selected *β* = 8. This value had the property that it produced a manageable network size (~ 130 genes), included > 25% of the seed node set, and that at least 80% of the solutions (even though based on computation with a heuristic component) were deemed optimal compared to exact analyses based on running the dapcst algorithm for each of the seed genes (Additional file 2: Figure S1).

Although this problem is NP-hard (nondeterministic polynomial time-hard) [31], the APCST algorithm is efficient in calculating exact and proximal solutions (DIMACs 11th challenge, http://dimacs11.zib.de/). The branch-and-bound algorithm, implemented in dapcst algorithm (https://github.com/mluipersbeck/dapcstp) and using default parameters, was used to find the optimal (or near optimal) APCST solution.

### Generation of networks using the dapcst algorithm

We used a particular variation of the ACPST (“root-ACPSTP”) where the search for the optimal solution starts in a specific node. This allowed us to force each seed node in turn to be included in the network, in contrast to the default APCST method which initialises network construction from the nodes with higher weights. For the main T2D analysis, therefore, the algorithm was run 451 times, once for each “seed” node. Runs generating a network of > 10 nodes (353 networks, median 155 nodes) were combined to form an ensemble network from the union of all 353 networks. This was reprojected onto the InWeb3 interactome to recover missing connections across nodes. As this final network represents a superposition of many different networks, linking nodes may sometimes appear at the periphery.

We assessed the specificity of each node in the final network by running the algorithm 100 times with the same parameter settings, but with random input data. We define specificity in this context as the complement of the percentage with which a given seed or linking node from the final network appears in runs generated from random input data. For each random run, we selected, from the InWeb3 interactome, random seed nodes matching the binding degree distribution of the observed set of seeds, and assigned them the same prize value as the original. Using the final parameter settings, we found that the included linking nodes were highly specific to our particular data, with 80% of them having a specificity higher than 75% (Additional file 3: Figure S2).

### Testing network for enrichment in the GWAS signal

To evaluate the extent to which the PPI network provided functional connectivity between positional candidates across loci, we measured the enrichment of the linking nodes for T2D association signals. This avoided the circularity of using co-expression or functional data to evaluate connectivity (as both contributed to the PCS determination). We generated gene-wise *p* values using the PASCAL method [32] from large-scale GWAS across a set of 33 traits (using data extracted from public repositories) including a recent meta-analysis of T2D GWAS data from ~ 150,000 Europeans [6]. We mapped these gene-wise association *p* values to linking nodes and converted them to *Z* scores using the standard normal cumulative distribution, *Z*_*i*_ = *ϕ*^−1^(1 − *p*_*i*_). We then quantified GWAS enrichment by aggregating the *Z* scores using Stouffer’s method:

$$ Zm\sim \frac{\sum \limits_{i=1}^k{Z}_i}{\sqrt{k}} $$

where *Z*_*i*_ is the *Z* score for the gene-wise *p* value for linking node *i* and *k* is the number of linking nodes in the network. Then, by permuting the InWeb3 network using a node permutation scheme, we compared the observed enrichment in GWAS signals to a random expectation, allowing us to calculate a nominal *p* value as:

$$ \left(\mathrm{Nominal}\ p\ \mathrm{value}\right)\ {p}_n=\frac{\#\left(Z> Zm\right)}{\#\left(\mathrm{total}\ \mathrm{permutations}\right)} $$

where *p*_*n*_ is the permuted *p* value generated in the permutation scheme. In this last step, the binding degree of all genes in the network is taken into full consideration (i.e. they all have the same binding degree as provided by the APCST network). To minimise bias arising from the co-localisation of genes with related functions (which is a feature of some parts of the genome), in each of these permutations, we only considered proteins whose genes mapped outside a 1-Mb window around the lead SNP for any significant GWAS association for that trait.

### APCST model clustering

To aid interpretation of the PPI networks, we used a community clustering algorithm that maximises network modularity and which breaks the full APCST model into smaller sub-networks [33].

### GTEx and islet RNA-Seq datasets

The InWeb3 PPI network we used is generated from empirically confirmed interactions but nevertheless includes many interactions that, owing to restricted tissue-specific expression, are unlikely to be biologically relevant. We used tissue-specific RNA expression data to filter the overall InWeb3 network and thereby generate in silico “tissue-specific” PPI networks, using TPM counts from GTEx (version 7: https://www.gtexportal.org/home/, last accessed 21 Oct 2017), complemented by human pancreatic islet data from [11]. Proteins with mRNA TPM counts < 0.1 in over 50% of samples for that tissue were removed from the InWeb network, allowing us to generate in silico PPI networks for 46 tissues. As an alternative strategy, we also attempted to generate tissue-specific PPI networks de novo from the subsets of genes transcribed in each tissue (as opposed to the strategy of filtering the overall PPI network). For the subset of tissues for which we generated such de novo tissue-specific networks, network content and topology was similar to that of the filtered approach (Jaccard index of similarity between 0.72 and 0.94 between paired analyses). For clarity and simplicity, we therefore used the filtered approach for all subsequent analyses described in the manuscript.

#### Functional enrichment analysis

Gene set enrichment (GSE) of networks and sub-networks was assessed with ClueGO [34] using GO terms and REACTOME gene sets [35]. The enrichment results were grouped using Cohen’s Kappa score of 0.4 and terms were considered significant when Bonferroni adjusted *p* value < 0.05 provided that there was an overlap of at least three network genes in the relevant GO gene set when calculating GO enrichment. For the pathway selection (REACTOME), we set a threshold that the network genes should represent at least 4% of the pathway. These values were applied given they are the recommended defaults when running ClueGO [34]. Cohen’s Kappa statistic measures the gene-set similarity of GO terms and REACTOME pathways and allowed us to group enriched terms into functional groups that improve visualisation of enriched pathways.

## Results

### Prioritising positional candidates at T2D risk loci

**Fig. 1:**
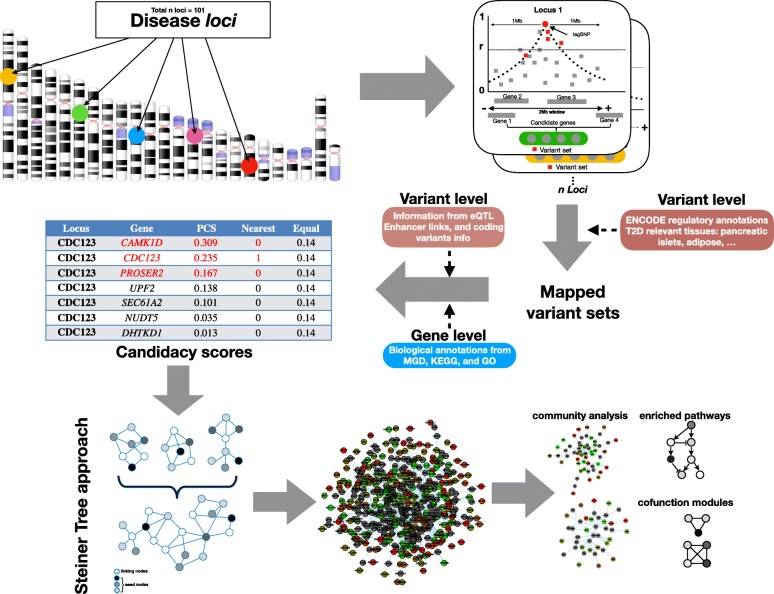
Overview of the data integration pipeline. We collected variants in the 1-Mb interval surrounding index variants at each of the 101 T2D GWAS loci along with relevant annotations for all protein coding genes in GENCODE including coding exon location, promoter location, distal regulatory elements correlated with gene activity from DNAseI hypersensitivity (DHS) data and summary statistic expression QTL (eQTL) data from T2D-relevant tissues. This, combined with information at the gene level from a semantic similarity metric, allowed us to define positional candidacy scores (PCS) for each gene in the GWAS intervals. PCS confers “weights” to the genes in the 1-Mb window based on biological candidacy, in contrast to “nearest” approaches, where the closest gene to the GWAS signal gets a weight of 1 and others a 0, or the “equal” approach where all genes in the window have the same weight. Genes with cumulative PCS > 0.7 were projected into the InWeb3 dataset using a Steiner tree algorithm to define a PPI network that maximises candidate gene connectivity. This network was further analysed to find processes, pathways and genes implicated in the T2D pathogenesis

We implemented a framework to derive positional candidacy scores (PCSs) for genes within T2D GWAS loci through the aggregation of two main types of data (Fig. 1; Methods). First, we used regression-based approaches to link disease-associated variants (most of which map to non-coding sequence and are therefore presumed to act through transcriptional regulation of nearby genes) to their likely effector transcripts, using a combination of variant-based annotations and expression QTL data.

Second, we scored each of the genes in these GWAS regions for disease-relevant biological function using semantic mapping of gene functional annotations from Gene Ontology, Mouse Genome Database and KEGG. We combined the evidence from both approaches, normalised across all genes at each GWAS locus, to generate the PCS for each gene.

We applied this method to score 1895 genes mapping within a 1-Mb interval around the lead variant at 101 T2D GWAS regions. This list of 101 T2D loci was assembled from a series of recent large-scale T2D GWAS [4, 6, 22] and represents the largest-effect T2D GWAS loci identified as of early 2017. The 1-Mb interval was selected to capture the majority of cis-acting regulatory effects (95% of cis-eQTLs map within 445 kb of the lead SNP [21]) and is therefore also likely to encompass most potential effector genes [36]. We observed only a weak correlation between the semantic and risk variant link scores for the 1895 positional candidates (*r*^2^ = 0.05, *p* = 0.01), indicating that these provide distinct information (Additional file 4: Figure S3).

Most (71%) of the 1895 genes had minimal evidence linking them to a causal role in T2D pathogenesis (PCS < 0.05) (Additional file 4: Figure S3). However, 95% of T2D loci included at least one gene (median, 3) with PCS > 0.10, and at 70% of loci, there was at least one gene with PCS > 0.20 (Additional file 4: Figure S3). The top-scoring genes across the 101 loci (such as *IRS1* [PCS = 0.69], *SLC30A8* [PCS = 0.77]*, HNF1B* [PCS = 0.54]) include many of the genes with the strongest prior claims for involvement in T2D risk, prior claims which arise in part from data used to generate the PCSs. For example, these genes each contain rare coding variants directly implicated in the development of T2D (or related conditions): these rare variants are independent of the common variant GWAS signals, but their relationship to diabetes is likely to have been captured through the semantic mapping. The PCS also highlighted several other highly scoring candidates with known causal roles in relation to diabetes and obesity such as *MC4R* (PCS = 0.43), *WFS1* (0.41), *ABCC8* (0.37), *LEP* (0.27), *GCK* (0.24) and *HNF1A* (0.23). At other loci, these analyses highlighted candidates that have received scant attention to date; for example, *CENPW* (PCS = 0.83) scored highly both in terms of semantic links to T2D-relevant processes and an adipose cis-eQTL linking the T2D GWAS SNP to *CENPW* expression [21].

To define the seed genes for subsequent PPI analyses, we gathered the sets of genes that, after ranking the transcripts for each locus by decreasing PCS, cumulatively accounted for at least 70% of the candidacy score for each locus. For example, at the *TP53INP1* locus, where the gene-specific PCSs range from 0.01 to 0.16 across a total of 17 mapped genes, the seed-gene set includes the first six (Additional file 5: Figure S4). This filter identified a total of 451 positional candidates across the loci, reducing the median number of genes per locus from 19 to 6 (Additional file 5: Figure S4). This filtering mostly removes genes with low PCS values: the proportion of genes with PCS < 0.05 falls from 71 to 12%, whilst most genes with PCS > 0.1 or > 0.2 are retained (Additional file 4: Figure S3).

This prioritisation process ensures that genes with the strongest combined causal evidence are favoured for network modelling, resulting in sets of seed genes that are more extensive than selection based on proximity alone (such as “nearest gene” approaches that seek to generate networks from only the genes mapping closest to the lead variants) but smaller than those which consider all regional genes of equal weight (“all gene” approaches). Note that our strategy does not require complete ascertainment of all true causal genes within this set of 451 genes: true effector genes excluded from the prioritised set of 451 genes (e.g. because they map more distal to the lead variant than 500 kb, or because low semantic scores reflect limited prior biological evidence) could still be available for “discovery” through the network modelling described below.

### Building a T2D-relevant protein-protein interactome

We set out to test whether this list of prioritised candidates could be used to characterise the functional relationships between genes (and proteins) implicated in T2D pathogenesis. Because the PCS used to prioritise the genes already incorporated (explicitly or otherwise) diverse types of functional and expression data, biasing any assessment of connectivity in these domains, we focused the network analysis around protein-protein interaction (PPI) data. To do so, we projected these 451 genes onto externally derived, empirically driven PPI resources (InWeb3) [19, 20] using an established network modelling strategy (the asymmetric prize-collecting Steiner tree (APCST)) (Fig. 1; Methods). In this analysis, the 451 positional candidates represent “seed” nodes which are used by the APCST algorithm to generate PPI networks which seek (with appropriate penalties to prevent frivolous propagation) to connect as many seed nodes as possible to each other, either directly, or using other (non-seed) proteins as links (“linking” nodes). The network topology is dependent only on the PCS values of the “seed” genes which are carried forward as weights into the APCST analysis, the confidence scores for each of the empirical PPI interactions in InWeb3, and the beta value used to tune the overall size of the PPI network generated (see Methods).

**Fig. 2:**
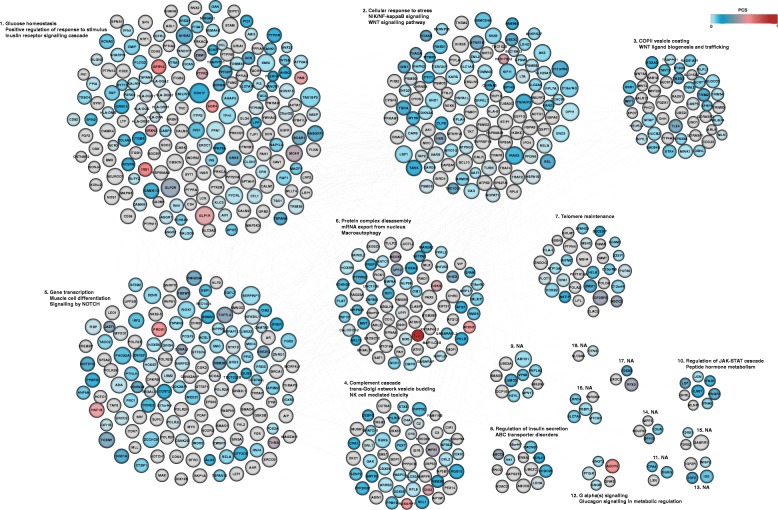
APCST final network. The final PPI network generated from the T2D GWAS interval genes includes 431 seed nodes and 274 linking nodes connected by 2678 interactions. We divided this network into 18 sub-networks (communities) using a community clustering algorithm that maximises network modularity [33], and highlighted enrichment of specific biological processes contained within these based on Gene Ontology terms and REACTOME pathways. Nodes are coloured according to their PCS with grey nodes representing linking nodes. Coloured nodes represent seed nodes, whereas grey nodes represent linking nodes. The size of the nodes is proportional to the network parameter neighbourhood connectivity. These networks are available in cytoscape and edge list format at [https://github.com/jfertaj/T2D_data_integration]

We operationalised the PPI network as follows (see Methods). Using each “seed” gene in turn, we used InWeb3 data to generate a PPI network that maximised the connectivity to other seed genes within the constraints of the APCST model. Of the 451 seed genes, 98 failed to produce a network exceeding 10 nodes. The remaining 353 networks had a median of 110 seeds and 45 linking nodes and were combined into an ensemble network, which was again projected into the InWeb3 interactome to recover missing connections between nodes. The final network contained 705 nodes (431 seed nodes, 274 linking nodes) and 2678 interactions (Fig. 2). Based on random networks generated with the same algorithm (see Methods), 80% of the linking nodes have a specificity for membership of the final network exceeding 75%, indicating that these linking nodes do not simply reflect generic hubs in the PPI space (Additional file 3: Figure S2).

### The T2D PPI network is enriched for T2D associations

If the final network truly provides novel insights into the functional relationships between genes thought to be mediating T2D predisposition, we reasoned that the “linking” genes (those brought into the network purely on the basis of external data indicating their protein-level interaction with seed genes) should be enriched for other seed gene characteristics. To avoid circularity arising from validation using data types that had contributed to the generation of the original PCS weights, including measures of gene function (e.g. GO, KEGG) or RNA expression data, we turned to T2D GWAS data, looking for empiric evidence that the genes encoding the linking proteins were themselves enriched for T2D association signals. For this, we used T2D association data from a set of ~ 150,000 European T2D case-control subjects imputed to 1000 Genomes [6]. Briefly, the linking nodes were mapped to gene-wise association *p* values generated from the GWAS results using PASCAL [32]. The significance of the collective enrichment of these gene-wise *p* values was obtained by permuting the observed set of linking nodes with equivalent sets of “random” nodes from the InWeb3 database, matched for binding degrees (see Methods). To minimise the prospects of picking up false signals arising from the combination of local LD and the non-random genomic location of functionally related genes, we excluded all genes from the 1-Mb window around the 101 lead variants from these analyses.

Compared to the distribution of scores in the permuted background, the gene-wise *p* values for linking genes in the empirical reconstructed network demonstrated significant enrichment of T2D association (*p* = 0.0014). To confirm that this enrichment was specific to T2D, we repeated the analysis, retaining the same PPI final network, but instead using GWAS data (and PASCAL-derived gene-wise *p* values) from 33 different traits across a wide range of disease areas. The only other traits displaying evidence of GWAS enrichment within the linking nodes of the T2D PPI network were those for anthropometric traits with known relevance to T2D pathophysiology (Additional file 6: Figure S5).

To gain insights into how the linking nodes of our final network contribute to T2D biology, we used the DisGeNET database [37], which collates gene-disease information from public data as well as from literature via natural language processing tools. We focused on the 274 linking nodes included in our model to avoid circularity arising from using the seeds, and identified 92 (~ 33%) with known links to T2D (Additional file 1: Table S2). Examples include as follows: (a) *NEUROD1* which encodes a transcription factor that is involved in the development of the endocrine cell lineage and has been implicated in monogenic diabetes [38], (b) *PRKCB* involved in insulin resistance [39] and (c) *GNAS*, implicated in beta-cell proliferation [40]. For this last gene, mouse knockouts have been shown to produce phenotypes concordant with diabetes [41]. These examples demonstrate the potential of these analyses to draw in “linking” nodes as related to T2D even when they are not located within genome-wide association signals.

### The T2D PPI network captures biological processes relevant to disease pathogenesis

To increase biological interpretability, we next sought to split the large final PPI network of 705 nodes into smaller sub-networks of closely interacting proteins (“communities”). Using the algorithm proposed by [33], we identified 18 such communities (each containing between 2 and 186 nodes) (Fig. 2). We performed enrichment analyses on each community using GO and REACTOME datasets, this time including both seed and linking nodes. We observed that the individual sub-networks were enriched for processes including “glucose homeostasis” and “insulin receptor signalling cascade” (sub-network 1), “Wnt” and “NIK/NF-kappaB signalling pathways” and “cellular response to stress” (sub-network 2), “COPII vesicle coating” and “Wnt ligand biogenesis and trafficking” (sub-network 3), “regulation of insulin secretion” (sub-network 8) and “glucagon signalling in metabolic regulation” (sub-network 12) (Fig. 2, Additional file 1: Table S3). This pattern of functional enrichment is broadly consistent with existing knowledge regarding aspects of T2D pathogenesis [42–44]. We saw no evidence in support of certain processes that have been proposed as contributors to T2D pathogenesis such as mitochondrial function or oxidative phosphorylation [45, 46], in line with the paucity of evidence linking these processes to T2D risk in standard gene-set enrichment analyses [4, 22].

### Information on tissue specificity enhances the model

The APCST model described above was constructed from a generic, tissue-agnostic PPI network. As a result, it features edges that, whilst they may be supported by the empirical data used to generate the InWeb3 database, are unlikely to be pathophysiologically relevant, due to mutually exclusive tissue-specific expression patterns. We hypothesised that the use of tissue-specific interactomes, focused on T2D-relevant tissues, would allow us to refine the reconstructed PPI network and might enhance the GWAS enrichment signal. In the absence of empirical PPI data for all relevant tissues, we generated these tissue-specific PPI networks by filtering on RNA transcript abundance. Starting from the generic final APCST network, we removed, for each tissue, all nodes (and their corresponding edges) with little or no transcriptional activity (see Methods). In all, we generated tissue-specific PPI networks, using RNA-Seq data sourced from 46 different tissues, 45 (including fat, liver and skeletal muscle) from GTEx (v7) [21][www.gtexportal.org] (median number of individuals = 235) together with a set of human islet RNA-Seq data (*n* = 118) [11], which had been reprocessed through a GTEx-aligned pipeline.

**Fig. 3:**
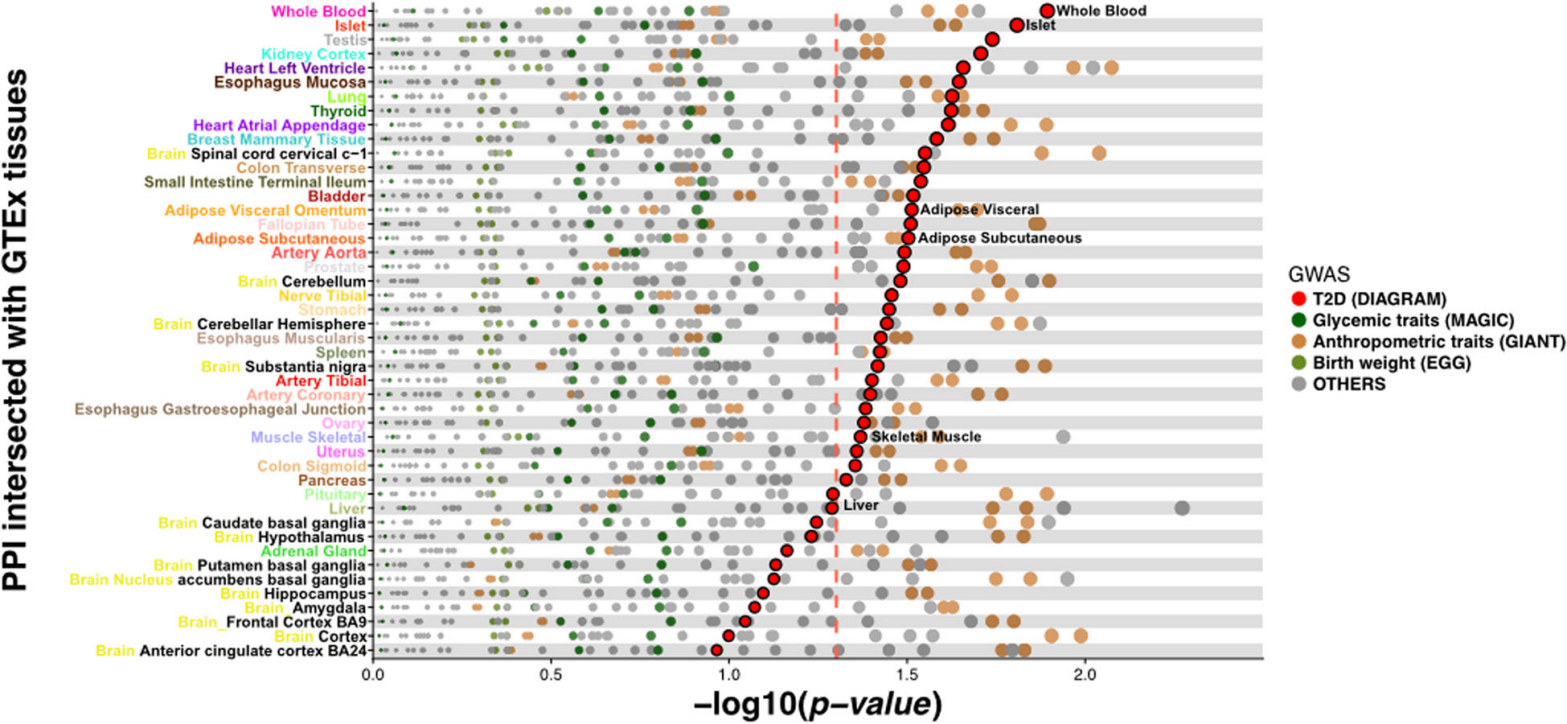
GWAS signal enrichment in tissue-specific interactomes. RNA-Seq data was used to filter the overall InWeb3 network and generate in silico tissue-specific networks that maximise connectivity between GWAS interval genes. Linking nodes within these networks were then tested for enrichment for GWAS signals using a permutation scheme. Each dot in the figure depicts the −log_10_ *p* value for enrichment for signals in a given GWAS dataset, for each of the 46 tissues. Dot colours reflect the GWAS phenotypes with T2D in the larger red colour. The dotted red line represents the nominal value of significance (*p* = 0.05), calculated as the empirical estimate of the null distribution from which the *p* value has been drawn. Islet showed the second strongest enrichment signal for T2D

We then repeated the T2D GWAS signal enrichment analysis (“linking” nodes only; 100,000 permutations) across each of these 46 tissue-specific PPI networks. We detected broad enrichment for T2D association in linking nodes across many of these tissue-specific networks: this likely reflects the fact that these tissue-specific networks remain highly overlapping (Additional file 7: Figure S6). Nonetheless, with the exception of whole blood, the strongest enrichment signal for T2D GWAS data was observed in the islet-specific PPI network (Fig. 3). This enrichment was less significant (*p* = 0.019) than that observed in the full network (*p* = 0.0014), but this, at least in part, reflects the reduction in the number of linking nodes in the islet-specific network (from 274 to 229). Other tissues implicated in T2D pathogenesis such as adipose, skeletal muscle or liver generated more limited evidence of enrichment (Fig. 3). This pattern of enrichment (favouring islets and, to a lesser degree, adipose) mirrors equivalent observations for other tissue-specific annotations (including cis-eQTL signals and active enhancers) with respect to T2D association data [10, 11].

### Further enhancement of model using GWAS locus subsets

**Fig. 4:**
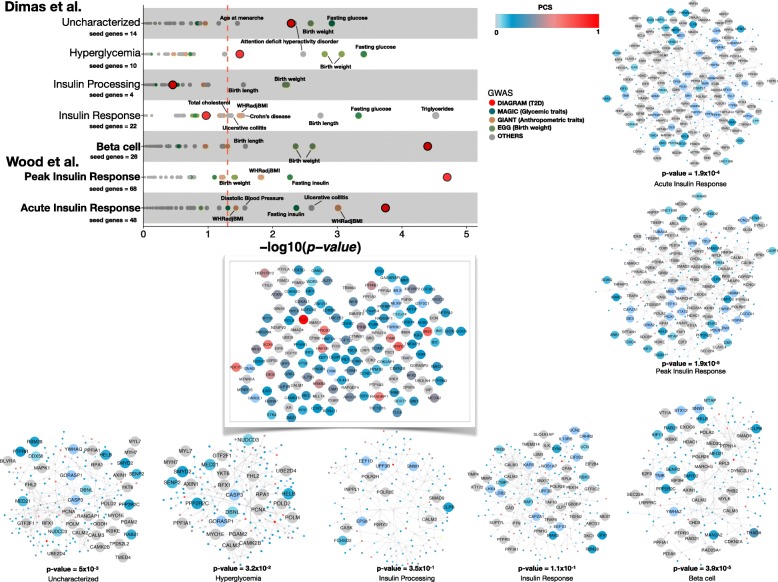
GWAS signal enrichment in an islet-specific network derived from T2D GWAS subsets. We built APCST networks filtered for islet RNA expression for each of the subsets of T2D GWAS loci defined by shared mechanistic mediation (refs [48, 49]). Number of seed genes for each of the subsets is displayed below the name of the phenotype. Enrichment in GWAS signals for linking nodes only was tested using a permutation scheme. Each dot in the figure depicts the −log_10_ *p* value of enrichment for association signals in a particular GWAS analysis. The results for T2D GWAS enrichment for the APCST networks built around the different T2D GWAS subsets are also represented (large red dots). The dotted red line represents nominal significance (*p* = 0.05) calculated as the empirical estimate of the null distribution from which the *p* value has been drawn. The strongest enrichment for T2D GWAS data in islet-filtered PPI data is observed for subsets of loci acting through reduced insulin secretion. The central network depicts the intersection of the seven networks created from the seven T2D GWAS locus subset categories. Nodes are coloured according to their PCS with grey nodes representing linking nodes. For the other networks, we have only named the nodes that are exclusive to each of the seven T2D GWAS locus subset category networks to ease interpretation. These networks are available in cytoscape and edge list format at [https://github.com/jfertaj/T2D_data_integration]

To further refine the analysis, we took account of the multi-organ nature of T2D and, specifically, of evidence that it is possible, using patterns of association across T2D-related quantitative traits such as BMI, lipids and insulin levels, to define subsets of T2D GWAS loci which impact primarily on insulin secretion and those that perturb insulin action [47–49]. We reasoned that the former would be expected to show preferential enrichment within the islet-filtered PPI network. Accordingly, we built APCST networks (both generic and filtered for expression in islets exactly as above) formed from the sets of high-PCS seed genes mapping to each of seven T2D GWAS locus subsets defined in two recent publications [48, 49] (Fig. 4; Additional file 8 : Figure S7).

In both the islet-specific (Fig. 4; Additional file 9: Figure S8) and the generic network (Additional file 8: Figure S7), the strongest signals for GWAS enrichment were seen for loci in the three subsets (beta cell [BC] in [48]; acute insulin response [AIR] and peak insulin response in [49]) comprised of T2D GWAS loci which influence T2D risk primarily through a detrimental effect on insulin secretion (Fig. 4; Additional file 9: Figure S8; Additional file 8: Figure S7; Additional file 10: Figure S9). In particular, there was striking enrichment in the islet-specific PPI network for linking nodes in the analyses of the BC (*p* = 3.9 × 10^− 5^) and AIR (*p* = 1.9 × 10^− 4^) T2D GWAS locus subsets.

As before, we were interested to see whether this marked convergence of PPI signal (as assessed by the enrichment of T2D association signals in linking nodes) was T2D-specific. We therefore repeated the enrichment analysis using GWAS data from 33 additional traits. For each trait, we took the APCST networks generated using the seven T2D locus subsets and assessed the “linking” nodes in those networks with respect to enrichment for respective gene-wise association *p* values. We found broad levels of enrichment for association signals for T2D-related phenotypes including (quantitative) glycemic traits, lipid levels, anthropometric and cardiovascular traits, which are consistent with known GWAS signal overlap. However, we saw very limited enrichment for other (non-diabetes related) traits. Furthermore, the patterns of enrichment were consistent with underlying physiological expectation: GWAS enrichment for anthropometric and lipid phenotypes was most marked in the APCST networks generated from the insulin-resistant subset of T2D loci (category “insulin response” in [48]), whilst T2D remained the most enriched phenotype for the subsets related to insulin secretion (Fig. 4; Additional file 10: Figure S9).

These analyses demonstrated that parallel efforts to refine the phenotypic impact of T2D GWAS loci, and the tissue-specificity of the underlying PPI dataset used to generate the APCST network, resulted in progressive, biologically appropriate, improvement of the enrichment signal observed at the “non-seed” proteins represented within the network.

### Validation and biological insights

To better understand the biological function of the highly enriched PPI network generated by the intersection of islet-specific expression, and the subset of T2D GWAS loci acting through reduced islet function (henceforth, the “islet network”), we performed a gene set enrichment analysis using GO and REACTOME terms (Additional file 1: Table S4). Captured pathways included well-known biological processes of “glucose homeostasis” (*p* = 1.5 × 10^− 4^), “regulation of WNT signalling pathway” (*p* = 8.9 × 10^− 3^), “response to insulin” (*p* = 6.2 × 10^− 4^) and “pancreas development” (*p* = 3.0 × 10^− 5^).

We also considered the extent to which it was possible to validate some of the genes highlighted within the network. The islet network identified many “seed” genes with a high T2D PCS (Fig. 4; Additional file 9: Figure S8) as candidate functional genes, including *SOX4* ([PCS = 0.62] at the locus usually named for *CDKAL1*) and *ATXN7* ([PCS = 0.57] at the locus named for *ADAMTS9*). In the former case, our prioritisation of *SOX4* has been validated by subsequent experimental studies which have demonstrated a direct relationship between perturbation of SOX4 and insulin secretion and beta-cell proliferation ([50, 51]).

Several other high-PCS candidate genes, such as *PAM* [0.54], *ADCY5* [0.63], *ZMIZ1* [0.22] or *THADA* [0.29], that were also included in the islet-specific network have also been validated by studies that (crucially) did not contribute to the original PCS determination. For example, the association signal near *PAM* has been convincingly localised to coding variants in *PAM* [5], which have been directly linked to defects in insulin content and secretion in human beta-cell models and islets [52]. At *ADCY5*, several groups ([10, 53]) have provided compelling experimental data that links the non-coding signals in the region to perturbation of the expression of *ADCY5* in islets, validating our assignment of candidacy.

A systematic assessment of the extent to which the network approach we have developed can robustly identify genes with a direct impact on the function of diabetes-relevant tissues, will have to await global perturbation studies (such as pooled CRISPR screens) that are currently underway. In the meantime, we looked for further examples of validation within the more limited perturbation screen performed by Thomsen and colleagues [13]. This analysis considered the phenotypic consequences, in a human beta cell line, of silencing approximately 300 genes which mapped to T2D GWAS regions that are largely overlapping with those considered in this paper. Selection of the 300 target genes was based on an earlier iteration of the PCS pipeline: consequently, the set is enriched for genes with a high PCS (median PCS of 0.18), limiting the extent to which the dataset could provide an independent readout of the relationship between PCS and function. Nevertheless, these data validate several more of our PCS assignments: for example, silencing of *ZMIZ1* was shown to significantly reduce insulin secretion, whilst *THADA* silencing has the opposite effect.

Some of the T2D GWAS loci we considered (e.g. *TLE4*, *CAGE1* and *GCK*) are represented by a single “seed” because the PCS for the highest-ranking gene exceeded 0.70. At other loci, this islet network does not include the gene with the highest PCS for the respective GWAS signal, but instead features an alternative gene from the same locus on the basis of its better connectivity within the network. Examples such as the gene *TBS* [PCS = 0.21] at the *ZBED3* locus, and *THRB* [PCS = 0.43] at the *UBE2E2* locus, demonstrate how the PPI data provides information additional to that used to derive the PCS. Neither of these genes was included in the published beta-cell screen [13], and further assessment of the functional impact of these and the other genes at these loci will be dependent on large-scale perturbation studies currently in progress.

Finally, several of the linking nodes introduced into this islet network through their PPI connections represent interesting candidates for a role in T2D pathogenesis, and there are several examples where external data provides validation of those assignments. An interesting example involves the gene *GINS4* which maps at the *ANK1* locus. Though this gene generated a low PCS [0.03] and was not included in the set of seed genes for this locus, *GINS4* knock-down has an impact in a human beta-cell line [14]. In addition, cyclin-dependent kinase 2 (*CDK2*) has been shown to influence beta-cell mass in a compensatory mechanism related to age- and diet-induced stress, connecting beta-cell dysfunction and progressive beta-cell mass deterioration [54]. YHWAG is a member of the 14-3-3 family, known to be signalling hubs for beta-cell survival [55], and disruption of *SMAD4* drives islet hypertrophy [56].

## Discussion

In this study, we set out to overcome two challenges that have impeded efforts to synthesise the biological information that is captured in the growing number of association signals emerging from GWAS. In the case of type 2 diabetes, for example, there are now well over a hundred independent common variant signals [6, 22], but most of these map to regulatory sequence, and the molecular mechanisms whereby these, individually and/or collectively, contribute to differences in T2D predisposition remain largely unresolved. A key question, of direct relevance to the opportunities for translational use of this information, is the extent to which, as the number of loci expands, there will be “saturation” or “convergence” of the biological mechanisms through which they operate, or whether, on the contrary, the range of networks and pathways implicated will continue to proliferate.

The first challenge concerns the identification of the effector transcripts through which the T2D predisposition effects at each of the GWAS signals (most obviously those that are regulatory) are mediated. We approached this challenge by integrating, for each of the genes within each of the GWAS signals, two types of data, one based around the fine-mapping of the causal variant, and the use of cis-eQTL data (in the case of regulatory variants) or direct coding variant inference to highlight the most likely effectors, and the other making use of diverse sources of biological information concerning the candidate effector genes and their protein products. Using this framework, we were able to assign candidacy scores to each regional gene, and then to deploy these scores as summaries of diverse sources of data that could be propagated into subsequent network analyses. Nevertheless, we recognise that, given the sparse nature of the data used, not all such candidacy assignments will be accurate, and the scaling of the PCSs at each locus means that candidacy scores are diluted at loci with high local gene density. However, these scores provide a principled and objective way of synthesising current knowledge, and the framework allows for iterative improvements in candidacy assignments as additional sources of relevant data become available. These are likely for example, to include further refinements in fine-mapping, additional links from associated variants to their effectors arising from chromatin conformation analyses, detection of rare coding variant signals through exome sequencing and genome-wide screens of transcript function.

The second challenge relates to the objective evaluation of the extent to which the strongest positional candidates at these GWAS loci occupy overlapping biological space. Standard approaches to network analysis applied to GWAS data—such as gene-set enrichment [32] or co-expression analyses—were not an option for this study since source data relevant to these had already been factored into the assessments of positional candidacy. Instead, we focused on the relationships between positional candidates as revealed by protein-protein interaction data, which we considered to be independent of the data in the earlier stages. We used the enrichment of T2D association signals in linking nodes (i.e. proteins included in the network which did not map to known GWAS loci) as our principal metric of network convergence.

## Conclusions

Applying our data integration approach, we were able to uncover a highly interconnected network associated with T2D, which was built around proteins involved in processes such as autophagy, lipid transport, cell growth and insulin receptor signalling pathways. We were able to show that this signal of enrichment was enhanced when we constrained the generic PPI network to reflect only genes expressed in pancreatic islets, and, concomitantly, limited the set of GWAS loci to those at which the T2D predisposition was mediated by defective islet function. These analyses reinforce the importance of the pancreatic islet as a critical tissue for the development of T2D and highlight multiple proteins (both those that map within GWAS loci, and those that fall outside) that are represented within this core islet network. These findings provide compelling hypotheses that can be explored further through direct experimental study, and also highlight the need to generate tissue-specific protein-protein interaction data. They also provide evidence to support a convergence of the mechanisms mediating predisposition across diverse T2D association signals.

Finally, these analyses demonstrate a valuable approach for the interrogation of large-scale GWAS data to capture biologically plausible disease-specific processes, one which can readily be applied to other complex diseases.

## Additional files

Additional file 1: **Table S1.** 101 loci and candidate genes by loci used to calculate the positional candidacy score (PCS). **Table S2.** DisGeNet results. **Table S3.** Gene set enrichment analysis by community. **Table S4.** Gene set enrichment analysis in beta cell islet-specific network. **Table S5.** List of genes from the seven T2D GWAS locus subset networks. (ZIP 360 kb)

Additional file 2: **Figure S1.** Correlation between β values and PPI network size. (TIFF 3072 kb)

Additional file 3: **Figure S2.** Specificity of linking nodes in the final network. (TIFF 3072 kb)

Additional file 4: **Figure S3.** Distribution of PCS and correlation of semantic and risk variant link scores. (TIFF 3072 kb)

Additional file 5: **Figure S4.** Summary of characteristics of PCS values. (PDF 200 kb)

Additional file 6: **Figure S5.** Enrichment of GWAS signals in the final PPI network. (TIFF 3072 kb)

Additional file 7: **Figure S6.** Correlations between tissue-specific PPI networks. (TIFF 3072 kb)

Additional file 8: **Figure S7.** GWAS signal enrichment in the PPI-generic network derived from T2D GWAs subsets. (TIFF 3072 kb)

Additional file 9: **Figure S8.** Sub-networks for seven T2D GWAS locus subset categories in the islet-specific network. (PDF 325 kb)

Additional file 10: **Figure S9.** Sub-networks for seven T2D GWAS locus subset categories in the PPI generic network. (PDF 380 kb)

## References

[1] International Diabetes Federation. IDF diabetes atlas, vol. 2017. 8th ed. Brussels: International Diabetes Federation; 2017.

[2] McCarthy MI (2010). Genomics, type 2 diabetes, and obesity. N Engl J Med.

[3] Agarwala V, Flannick J, Sunyaev S, Go TDC, Altshuler D (2013). Evaluating empirical bounds on complex disease genetic architecture. Nat Genet.

[4] Fuchsberger C, Flannick J, Teslovich TM, Mahajan A, Agarwala V, Gaulton KJ (2016). The genetic architecture of type 2 diabetes. Nature..

[5] Mahajan A, Wessel J, Willems SM, Zhao W, Robertson NR, Chu AY (2018). Refining the accuracy of validated target identification through coding variant fine-mapping in type 2 diabetes. Nat Genet.

[6] Scott RA, Scott LJ, Magi R, Marullo L, Gaulton KJ, Kaakinen M (2017). An expanded genome-wide association study of type 2 diabetes in Europeans. Diabetes..

[7] Boyle EA, Li YI, Pritchard JK (2017). An expanded view of complex traits: from polygenic to omnigenic. Cell..

[8] Gaulton KJ, Ferreira T, Lee Y, Raimondo A, Magi R, Reschen ME (2015). Genetic fine mapping and genomic annotation defines causal mechanisms at type 2 diabetes susceptibility loci. Nat Genet.

[9] Varshney A, Scott LJ, Welch RP, Erdos MR, Chines PS, Narisu N (2017). Genetic regulatory signatures underlying islet gene expression and type 2 diabetes. Proc Natl Acad Sci U S A.

[10] Thurner M, van de Bunt M, Torres JM, Mahajan A, Nylander V, Bennett AJ, et al. Integration of human pancreatic islet genomic data refines regulatory mechanisms at type 2 diabetes susceptibility loci. Elife. 2018;7:e31977. 10.7554/eLife.31977 PMC5828664 29412141

[11] van de Bunt M, Manning Fox JE, Dai X, Barrett A, Grey C, Li L (2015). Transcript expression data from human islets links regulatory signals from genome-wide association studies for type 2 diabetes and glycemic traits to their downstream effectors. PLoS Genet.

[12] Hughes JR, Roberts N, McGowan S, Hay D, Giannoulatou E, Lynch M (2014). Analysis of hundreds of cis-regulatory landscapes at high resolution in a single, high-throughput experiment. Nat Genet.

[13] Thomsen SK, Ceroni A, van de Bunt M, Burrows C, Barrett A, Scharfmann R (2016). Systematic functional characterization of candidate causal genes for type 2 diabetes risk variants. Diabetes..

[14] Thomsen SK, Gloyn AL (2014). The pancreatic beta cell: recent insights from human genetics. Trends Endocrinol Metab.

[15] Kohler S, Bauer S, Horn D, Robinson PN (2008). Walking the interactome for prioritization of candidate disease genes. Am J Hum Genet.

[16] Lee SA, Tsao TT, Yang KC, Lin H, Kuo YL, Hsu CH (2011). Construction and analysis of the protein-protein interaction networks for schizophrenia, bipolar disorder, and major depression. BMC Bioinformatics.

[17] Hou L, Chen M, Zhang CK, Cho J, Zhao H (2014). Guilt by rewiring: gene prioritization through network rewiring in genome wide association studies. Hum Mol Genet.

[18] Lundby A, Rossin EJ, Steffensen AB, Acha MR, Newton-Cheh C, Pfeufer A (2014). Annotation of loci from genome-wide association studies using tissue-specific quantitative interaction proteomics. Nat Methods.

[19] Lage K, Karlberg EO, Storling ZM, Olason PI, Pedersen AG, Rigina O (2007). A human phenome-interactome network of protein complexes implicated in genetic disorders. Nat Biotechnol.

[20] Rossin EJ, Lage K, Raychaudhuri S, Xavier RJ, Tatar D, Benita Y (2011). Proteins encoded in genomic regions associated with immune-mediated disease physically interact and suggest underlying biology. PLoS Genet.

[21] The GTEx Consortium. Genetic effects on gene expression across human tissues. Nature. 2017;550:204. 10.1038/nature24277 PMC5776756 29022597

[22] Morris AP, Voight BF, Teslovich TM, Ferreira T, Segre AV, Steinthorsdottir V (2012). Large-scale association analysis provides insights into the genetic architecture and pathophysiology of type 2 diabetes. Nat Genet.

[23] Harrow J, Frankish A, Gonzalez JM, Tapanari E, Diekhans M, Kokocinski F (2012). GENCODE: the reference human genome annotation for the ENCODE project. Genome Res.

[24] Consortium TEP (2012). An integrated encyclopedia of DNA elements in the human genome. Nature..

[25] Mahajan A, Go MJ, Zhang W, Below JE, Gaulton KJ, Ferreira T (2014). Genome-wide trans-ancestry meta-analysis provides insight into the genetic architecture of type 2 diabetes susceptibility. Nat Genet.

[26] Steinthorsdottir V, Thorleifsson G, Sulem P, Helgason H, Grarup N, Sigurdsson A (2014). Identification of low-frequency and rare sequence variants associated with elevated or reduced risk of type 2 diabetes. Nat Genet.

[27] Wild F (2005). lsa: Latent Semantic Analysis. R Package Version 0.57 ed.

[28] Dittrich MT, Klau GW, Rosenwald A, Dandekar T, Muller T (2008). Identifying functional modules in protein-protein interaction networks: an integrated exact approach. Bioinformatics..

[29] Tuncbag N, McCallum S, Huang SS, Fraenkel E (2012). SteinerNet: a web server for integrating ‘omic’ data to discover hidden components of response pathways. Nucleic Acids Res.

[30] Balbin OA, Prensner JR, Sahu A, Yocum A, Shankar S, Malik R (2013). Reconstructing targetable pathways in lung cancer by integrating diverse omics data. Nat Commun.

[31] Garey MR, Johnson DS (1979). Computers and intractability : a guide to the theory of NP-completeness.

[32] Lamparter D, Marbach D, Rueedi R, Kutalik Z, Bergmann S (2016). Fast and rigorous computation of gene and pathway scores from SNP-based summary statistics. PLoS Comput Biol.

[33] Clauset A, Newman ME, Moore C (2004). Finding community structure in very large networks. Phys Rev E Stat Nonlinear Soft Matter Phys.

[34] Bindea G, Mlecnik B, Hackl H, Charoentong P, Tosolini M, Kirilovsky A (2009). ClueGO: a Cytoscape plug-in to decipher functionally grouped gene ontology and pathway annotation networks. Bioinformatics..

[35] Croft D, Mundo AF, Haw R, Milacic M, Weiser J, Wu G (2014). The Reactome pathway knowledgebase. Nucleic Acids Res.

[36] Shin SY, Fauman EB, Petersen AK, Krumsiek J, Santos R, Huang J (2014). An atlas of genetic influences on human blood metabolites. Nat Genet.

[37] Pinero J, Bravo A, Queralt-Rosinach N, Gutierrez-Sacristan A, Deu-Pons J, Centeno E (2017). DisGeNET: a comprehensive platform integrating information on human disease-associated genes and variants. Nucleic Acids Res.

[38] Rubio-Cabezas O, Minton JA, Kantor I, Williams D, Ellard S, Hattersley AT (2010). Homozygous mutations in NEUROD1 are responsible for a novel syndrome of permanent neonatal diabetes and neurological abnormalities. Diabetes..

[39] Yuan W, Xia Y, Bell CG, Yet I, Ferreira T, Ward KJ (2014). An integrated epigenomic analysis for type 2 diabetes susceptibility loci in monozygotic twins. Nat Commun.

[40] Kimple ME, Moss JB, Brar HK, Rosa TC, Truchan NA, Pasker RL (2012). Deletion of GalphaZ protein protects against diet-induced glucose intolerance via expansion of beta-cell mass. J Biol Chem.

[41] Weinstein MM, Goulbourne CN, Davies BS, Tu Y, Barnes RH, Watkins SM (2012). Reciprocal metabolic perturbations in the adipose tissue and liver of GPIHBP1-deficient mice. Arterioscler Thromb Vasc Biol.

[42] Bergman BC, Cornier MA, Horton TJ, Bessesen DH (2007). Effects of fasting on insulin action and glucose kinetics in lean and obese men and women. Am J Physiol Endocrinol Metab.

[43] Bano G (2013). Glucose homeostasis, obesity and diabetes. Best Pract Res Clin Obstet Gynaecol.

[44] Arnold AC, Robertson D (2015). Defective Wnt signaling: a potential contributor to cardiometabolic disease?. Diabetes..

[45] Wang CH, Wang CC, Wei YH (2010). Mitochondrial dysfunction in insulin insensitivity: implication of mitochondrial role in type 2 diabetes. Ann N Y Acad Sci.

[46] Antoun G, McMurray F, Thrush AB, Patten DA, Peixoto AC, Slack RS (2015). Impaired mitochondrial oxidative phosphorylation and supercomplex assembly in rectus abdominis muscle of diabetic obese individuals. Diabetologia..

[47] Voight BF, Scott LJ, Steinthorsdottir V, Morris AP, Dina C, Welch RP (2010). Twelve type 2 diabetes susceptibility loci identified through large-scale association analysis. Nat Genet.

[48] Dimas AS, Lagou V, Barker A, Knowles JW, Magi R, Hivert MF (2014). Impact of type 2 diabetes susceptibility variants on quantitative glycemic traits reveals mechanistic heterogeneity. Diabetes..

[49] Wood AR, Jonsson A, Jackson AU, Wang N, van Leewen N, Palmer ND (2017). A genome-wide association study of IVGTT-based measures of first-phase insulin secretion refines the underlying physiology of type 2 diabetes variants. Diabetes..

[50] Collins SC, Do HW, Hastoy B, Hugill A, Adam J, Chibalina MV (2016). Increased expression of the diabetes gene SOX4 reduces insulin secretion by impaired fusion pore expansion. Diabetes..

[51] Xu EE, Sasaki S, Speckmann T, Nian C, Lynn FC (2017). SOX4 allows facultative beta-cell proliferation through repression of Cdkn1a. Diabetes..

[52] Thomsen SK, Raimondo A, Hastoy B, Sengupta S, Dai XQ, Bautista A (2018). Type 2 diabetes risk alleles in PAM impact insulin release from human pancreatic beta-cells. Nat Genet.

[53] Roman TS, Cannon ME, Vadlamudi S, Buchkovich ML, Wolford BN, Welch RP (2017). A type 2 diabetes-associated functional regulatory variant in a pancreatic islet enhancer at the ADCY5 locus. Diabetes..

[54] Kim SY, Lee JH, Merrins MJ, Gavrilova O, Bisteau X, Kaldis P (2017). Loss of cyclin-dependent kinase 2 in the pancreas links primary beta-cell dysfunction to progressive depletion of beta-cell mass and diabetes. J Biol Chem.

[55] Lim GE, Piske M, Johnson JD (2013). 14-3-3 proteins are essential signalling hubs for beta cell survival. Diabetologia..

[56] Simeone DM, Zhang L, Treutelaar MK, Zhang L, Graziano K, Logsdon CD (2006). Islet hypertrophy following pancreatic disruption of Smad4 signaling. Am J Physiol Endocrinol Metab.

