# Supplementary material for: Developing a network view of type 2 diabetes risk pathways through integration of genetic, genomic and functional data
Source: Genome Med. 2019 Mar 26;11:19. doi: 10.1186/s13073-019-0628-8 (PMC6436236; doi:10.1186/s13073-019-0628-8)
Supplement: Supplementary file 1 — Table S1. 101 loci and candidate genes by loci used to calculate the positional candidacy score (PCS). Table S2. DisGeNet results. Table S3. Gene set enrichment analysis by community. Table S4. Gene set enrichment analysis in beta cell islet-specific network. Table S5. List of genes from the seven T2D GWAS locus subset networks. (ZIP 360 kb) [file 13073_2019_628_MOESM1_ESM.zip › Additonal_file_1_tables.docx]

**S1 Table. 101 loci and candidate genes by loci used to calculate the Positional Candidacy Score (PCS)**. Note: We developed a framework to score the candidacy of genes mapping to GWAS association signals which aggregated data from multiple sources. The information collected fell into two categories. First, we used regression-based approaches to link disease-associated variants to their likely effector transcripts, using a combination of variant-based annotations and expression QTL data (Link score). Second, we scored each of the genes in these GWAS regions for disease-relevant biological function (Semantic score).

For naming the loci (column Locus) we have used the convention of using one of the included genes in that region, usually the one nearest the association peak. We combined the two measures to generate a “positional candidacy score” (PCS) for each gene. Cumulative: cumulative frequency of PCS for each locus. References: Bibliographic references describing the loci as associated to type II diabetes.

**S2 Table. DisGeNet results**. MeSH = Medical Subject Headings; DPI score = disease pleiotropic index; DSI score = disease specific index; GDA Score = Gene-Disease Association Score; EI = Evidence Score.

**S3 Table. Gene Set Enrichment Analysis by community**. GOID: Gene Ontology ID; GOTerm: Gene Ontology Term; Note: Gene Set Enrichment (GSE) of networks and sub-networks was performed with ClueGO using GO terms and REACTOME gene sets. The enrichment results were considered significant when bonferroni adjusted p-value < 0.05 and at least 3% of the genes contained in the tested gene set is included in the network. Gene sets were also grouped using kappa score into functional groups to improve visualization of enriched pathways.

**S4 Table. Gene Set Enrichment Analysis in Beta-cell Islet-specific network**. GOID: Gene Ontology ID; GOTerm: Gene Ontology Term. Note: Gene Set Enrichment (GSE) of networks and sub-networks was performed with ClueGO using GO terms and REACTOME gene sets. The enrichment results were considered significant when bonferroni adjusted p-value < 0.05 and at least 3% of the genes contained in the tested gene set is included in the network. Gene sets were also grouped using kappa score into functional groups to improve visualization of enriched pathways.

**S5 Table. List of genes from the seven T2D GWAS locus subset networks.**

This table shows the genes that form the seven T2D GWAS locus subset networks from Figure 4 and supplementary figure 7.
