# Supplementary figures and images for: Developing a network view of type 2 diabetes risk pathways through integration of genetic, genomic and functional data
Source: Genome Med. 2019 Mar 26;11:19. doi: 10.1186/s13073-019-0628-8 (PMC6436236; doi:10.1186/s13073-019-0628-8)

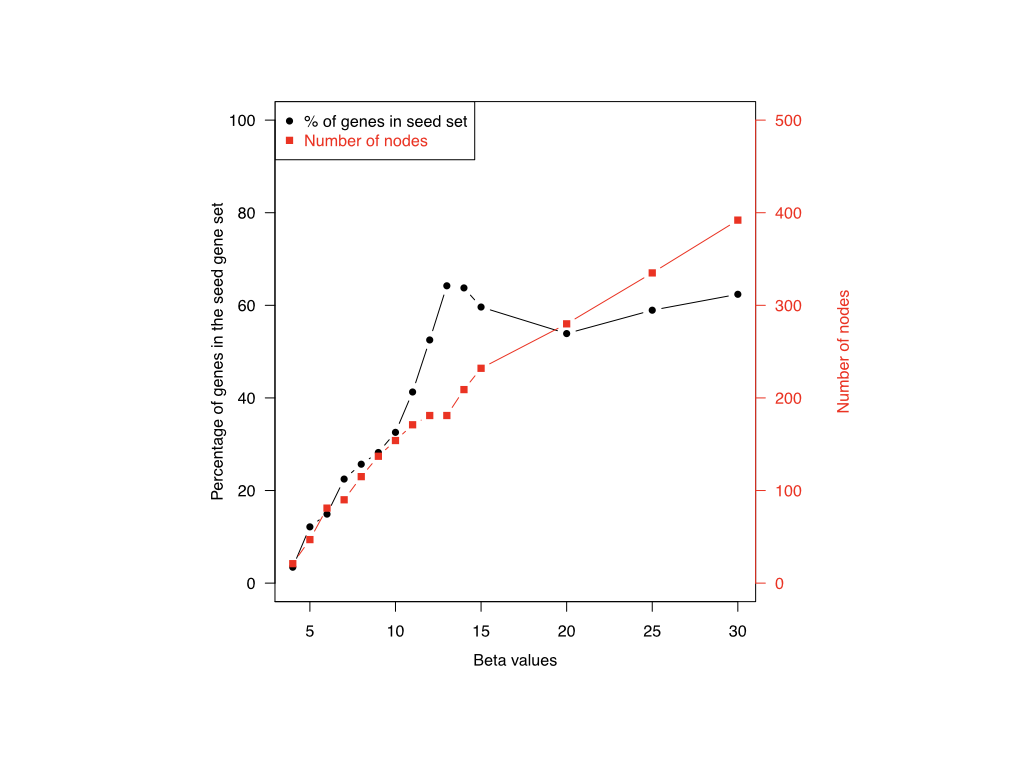

Supplement: Supplementary file 2 — Figure S1. Correlation between β values and PPI network size. (TIFF 3072 kb) [file 13073_2019_628_MOESM2_ESM.tiff]

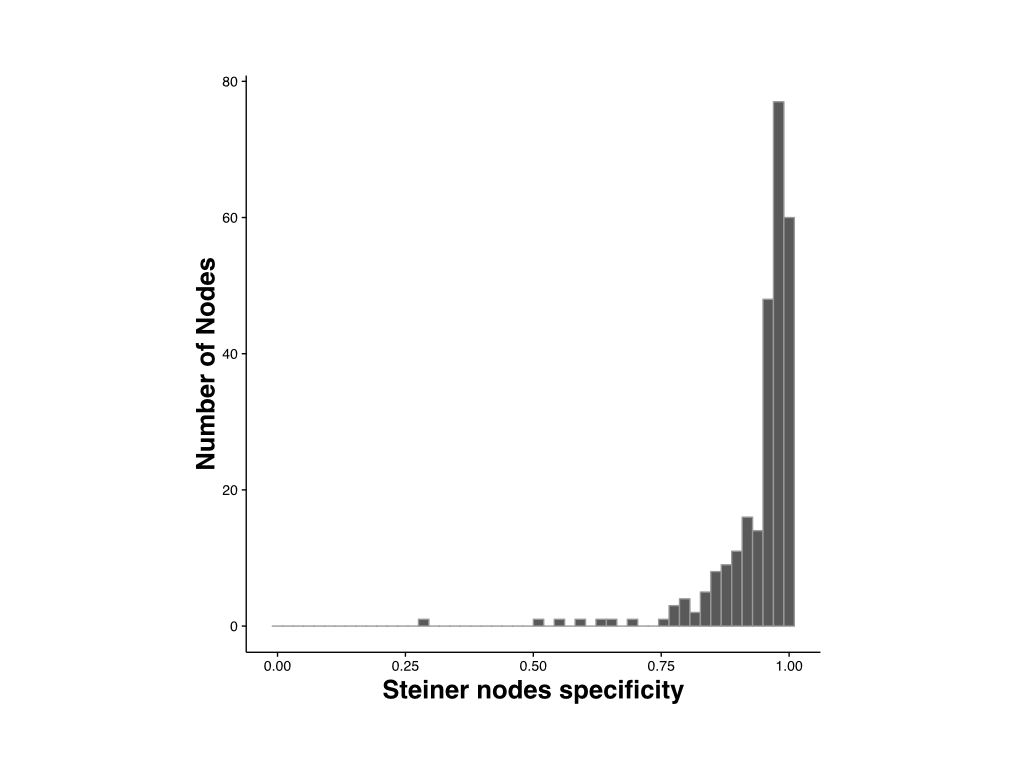

Supplement: Supplementary file 3 — Figure S2. Specificity of linking nodes in the final network. (TIFF 3072 kb) [file 13073_2019_628_MOESM3_ESM.tiff]

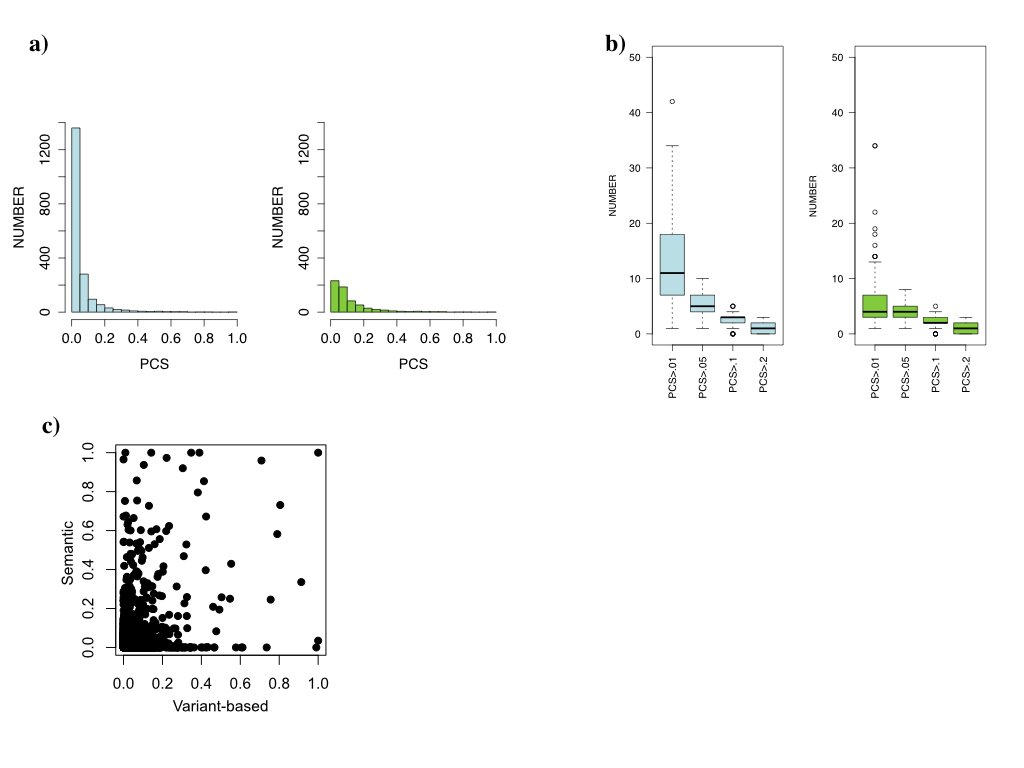

Supplement: Supplementary file 4 — Figure S3. Distribution of PCS and correlation of semantic and risk variant link scores. (TIFF 3072 kb) [file 13073_2019_628_MOESM4_ESM.tiff]

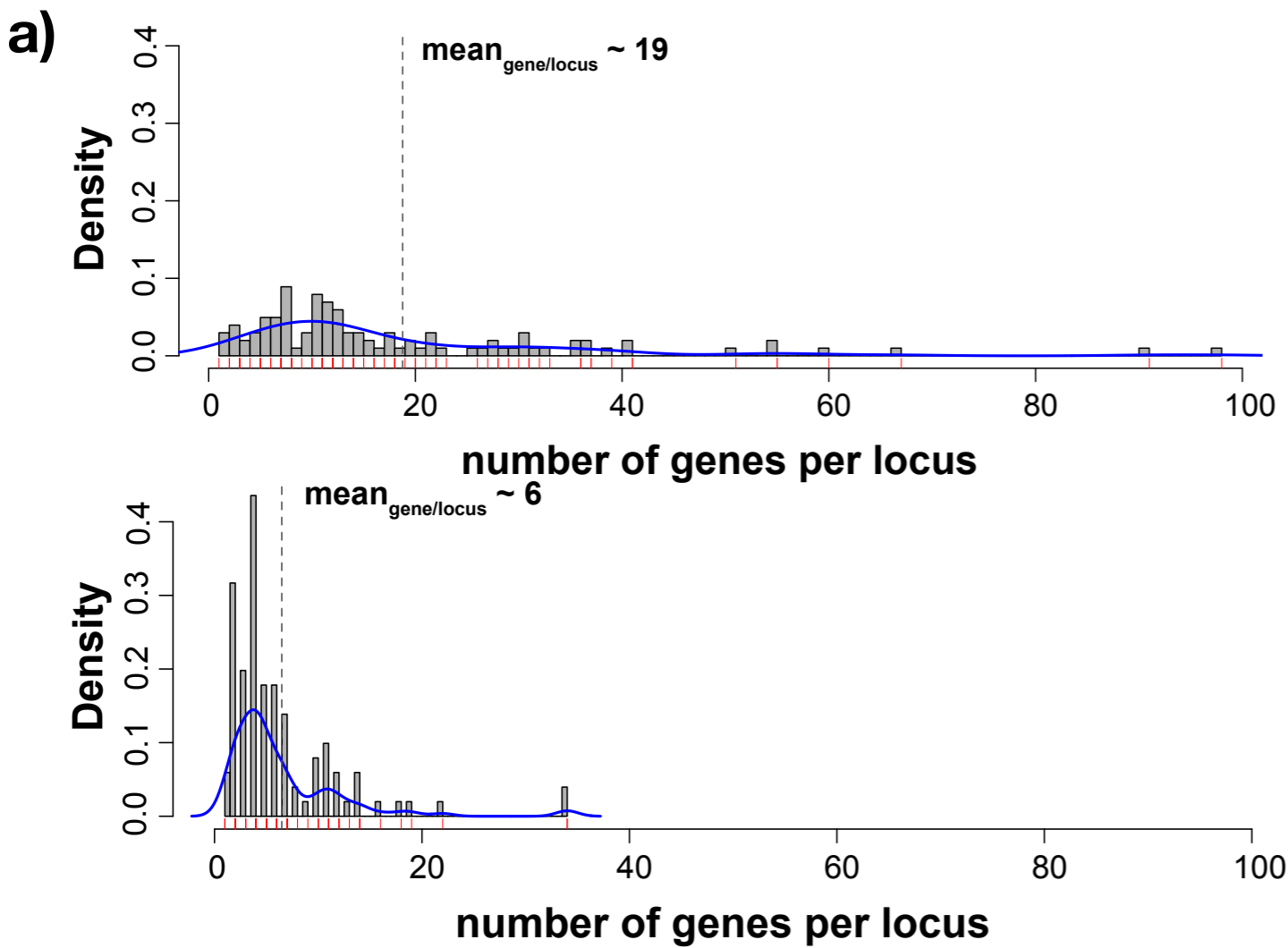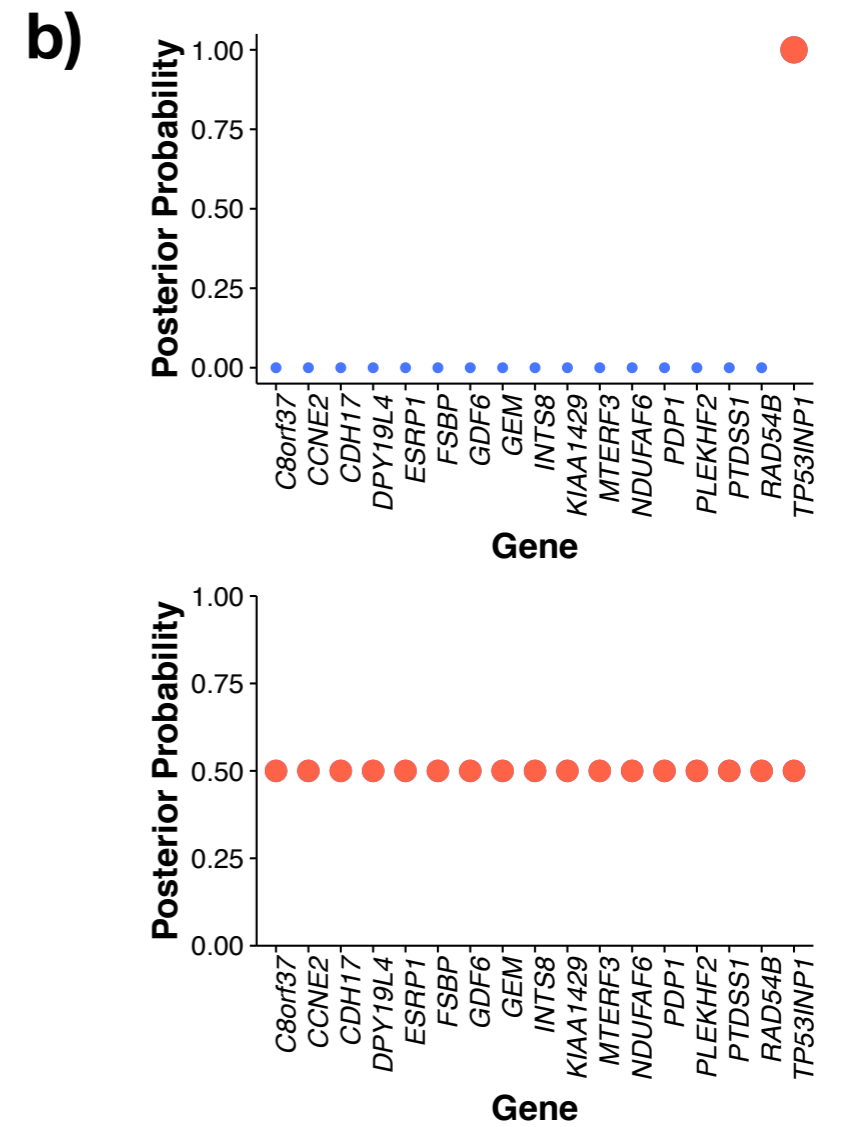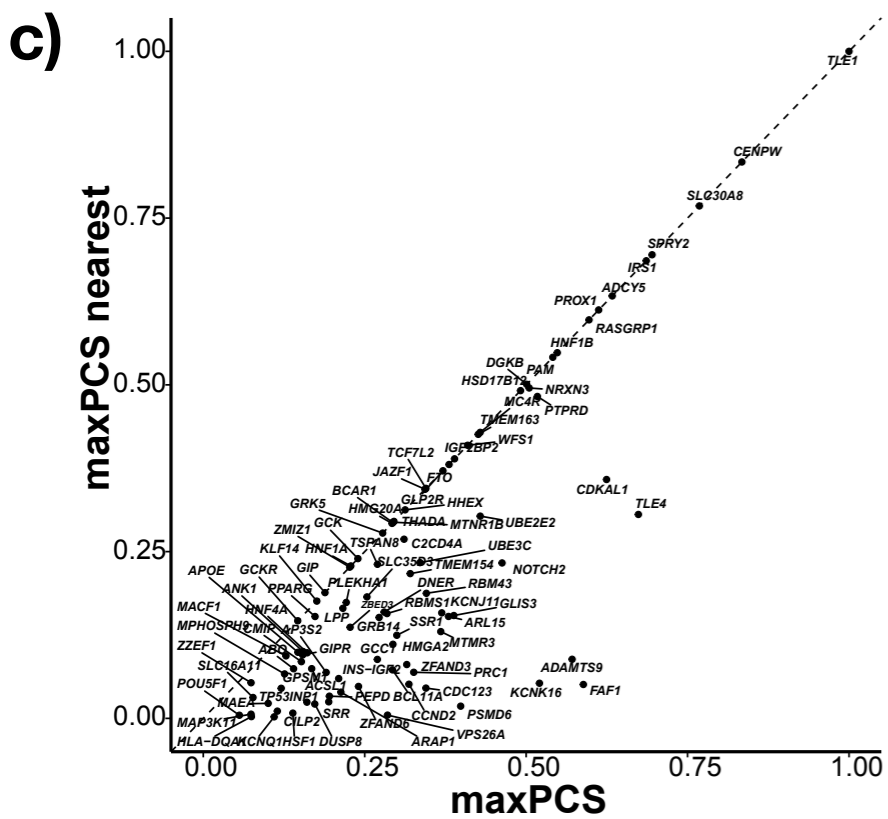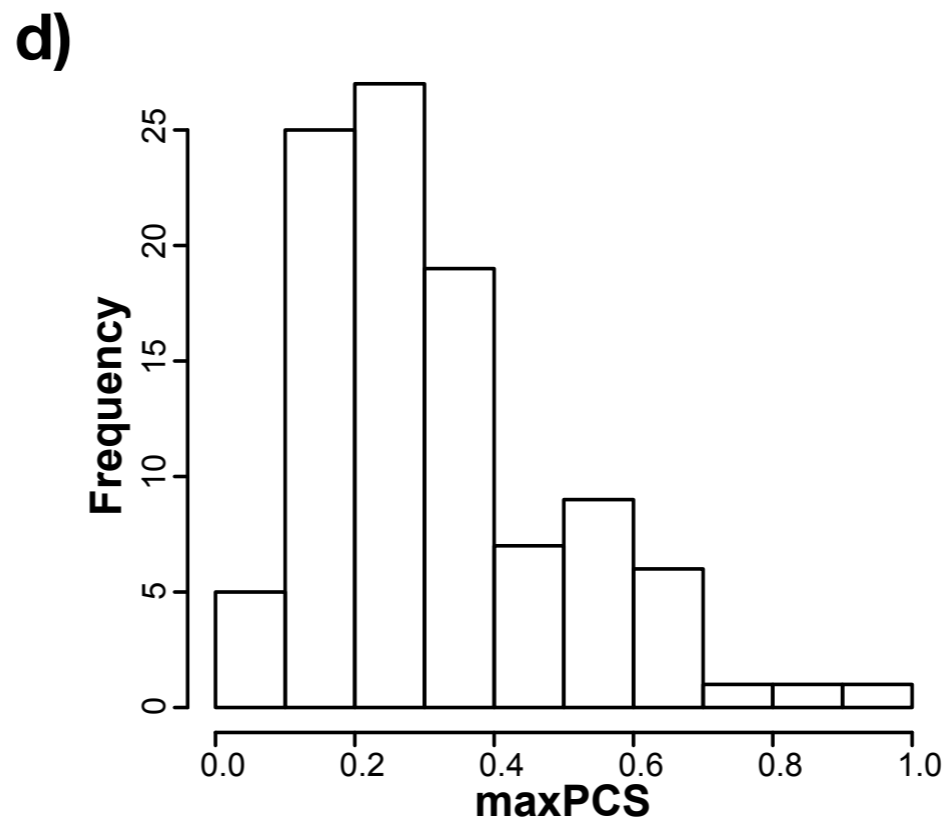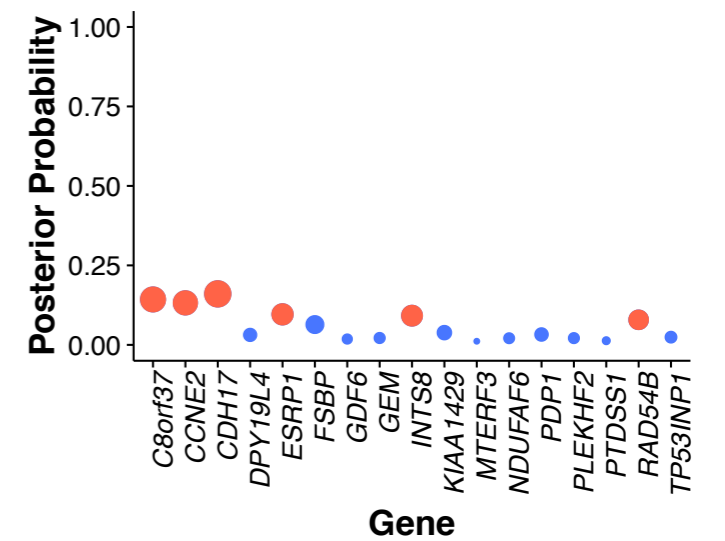

Supplement: Supplementary file 5 — Figure S4. Summary of characteristics of PCS values. (PDF 200 kb) [file 13073_2019_628_MOESM5_ESM.pdf]

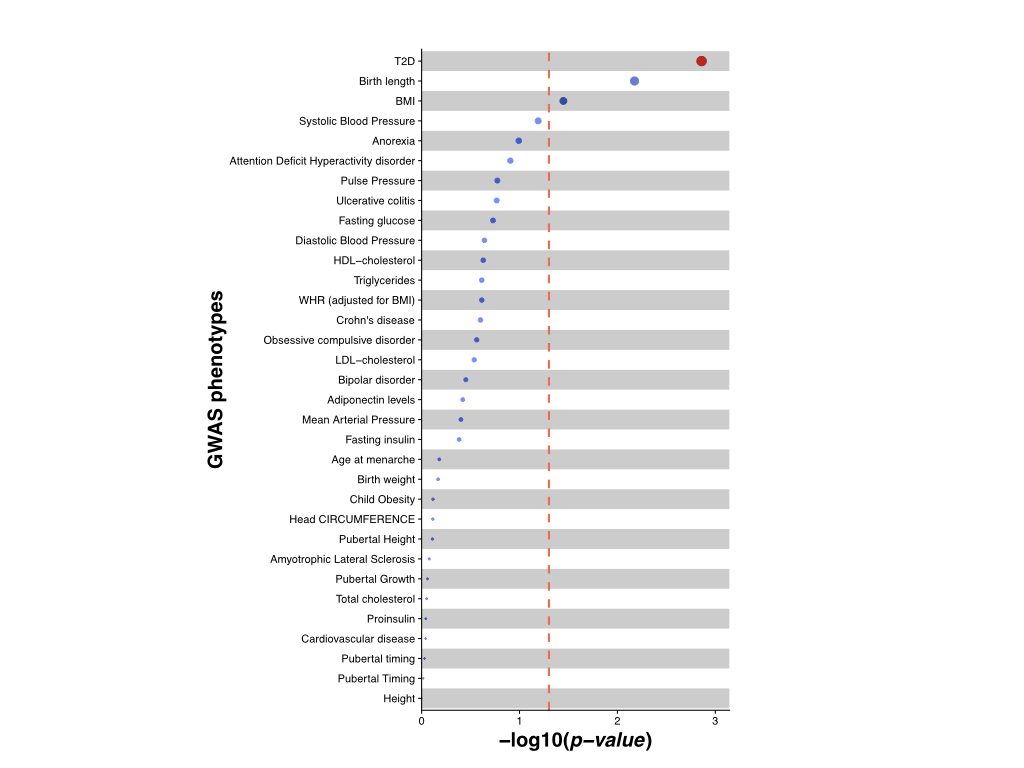

Supplement: Supplementary file 6 — Figure S5. Enrichment of GWAS signals in the final PPI network. (TIFF 3072 kb) [file 13073_2019_628_MOESM6_ESM.tiff]

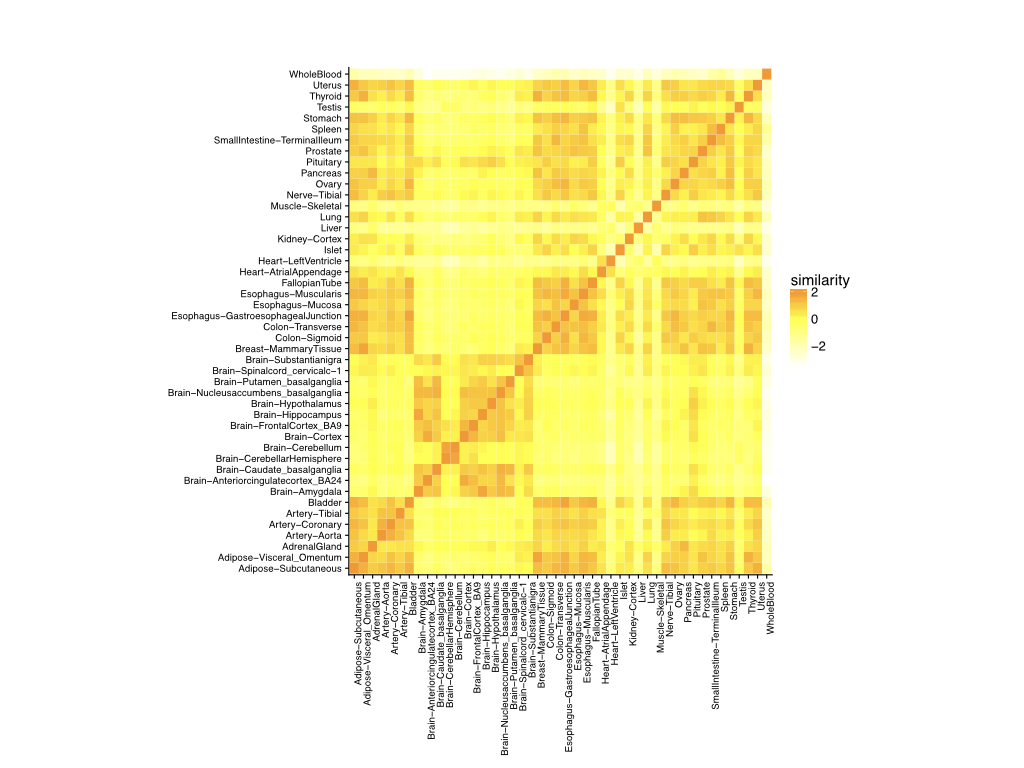

Supplement: Supplementary file 7 — Figure S6. Correlations between tissue-specific PPI networks. (TIFF 3072 kb) [file 13073_2019_628_MOESM7_ESM.tiff]

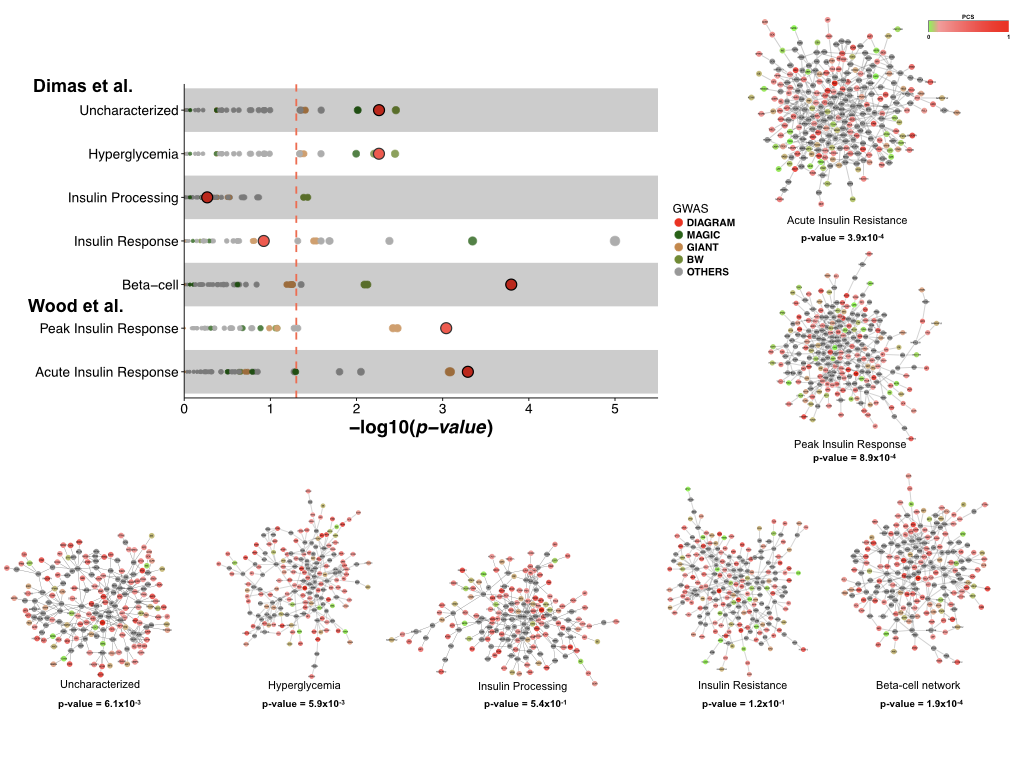

Supplement: Supplementary file 8 — Figure S7. GWAS signal enrichment in the PPI-generic network derived from T2D GWAs subsets. (TIFF 3072 kb) [file 13073_2019_628_MOESM8_ESM.tiff]

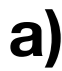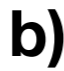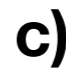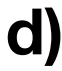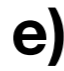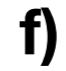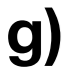

Supplement: Supplementary file 10 — Figure S9. Sub-networks for seven T2D GWAS locus subset categories in the PPI generic network. (PDF 380 kb) [file 13073_2019_628_MOESM10_ESM.pdf]
