# Supplementary material for: Developing a network view of type 2 diabetes risk pathways through integration of genetic, genomic and functional data
Source: Genome Med. 2019 Mar 26;11:19. doi: 10.1186/s13073-019-0628-8 (PMC6436236; doi:10.1186/s13073-019-0628-8)
Supplement: Supplementary file 9 — Figure S8. Sub-networks for seven T2D GWAS locus subset categories in the islet-specific network. (PDF 325 kb) [file 13073_2019_628_MOESM9_ESM.pdf]

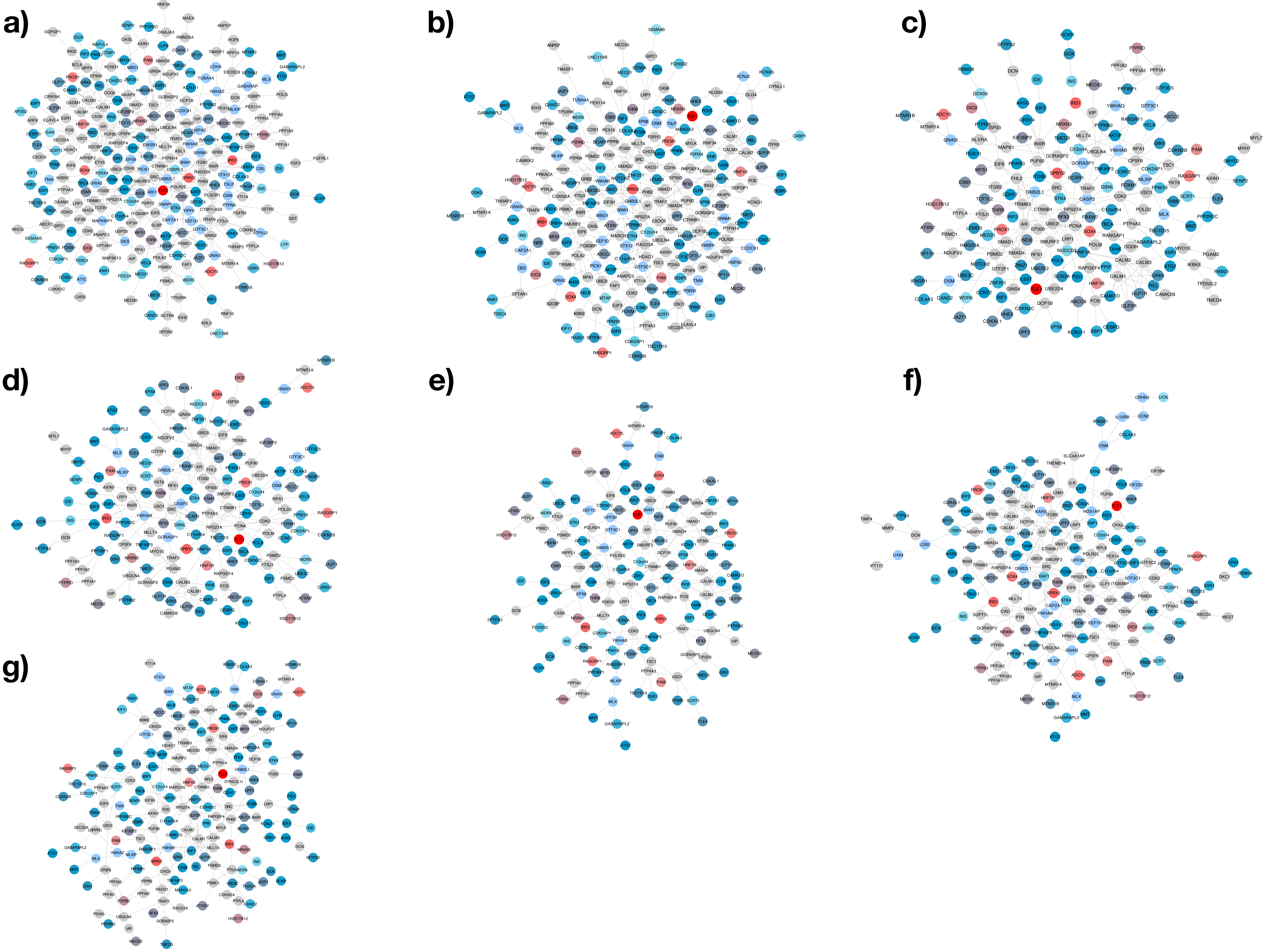
a)
b)
c)
d)
e)
f)
g)
